# Supplementary material for: Quantity, Duration, Adherence, and Reasons for Dietary Supplement Use among Adults: Results from NHANES 2011–2018
Source: Nutrients. 2024 Jun 11;16(12):1830. doi: 10.3390/nu16121830 (PMC11206876; doi:10.3390/nu16121830)
Supplement: Supplementary file 1 [file nutrients-16-01830-s001.zip › nutrients-3002975-supplementary.pdf]

Supplemental Table S1. Reasons for using dietary supplements hand card

**To:**
Build muscle
Gain weight
Get more energy
Improve digestion
Improve my overall health
Maintain health (to stay healthy)
Maintain healthy blood sugar level, diabetes
Prevent colds, boost immune system
Prevent health problems
Supplement my diet (because I don't get enough from food)
**For:**
Anemia, such as low iron
Bone health, build strong bones, osteoporosis
Eye health
Good bowel/colon health
Healthy Joints, arthritis
Healthy skin, hair, and nails
Heart health, cholesterol
Kidney and bladder health, urinary tract health
Liver health, detoxification, cleanse system
Menopause, hot flashes
Mental health
Muscle related issues, muscle cramps
Pregnancy/breastfeeding
Prostate health
Relaxation, decrease stress, improve sleep
Teeth, prevent cavities
Weight loss
Inflammation
Fluid/water balance

Supplemental Figure S1. Flowchart of selection process

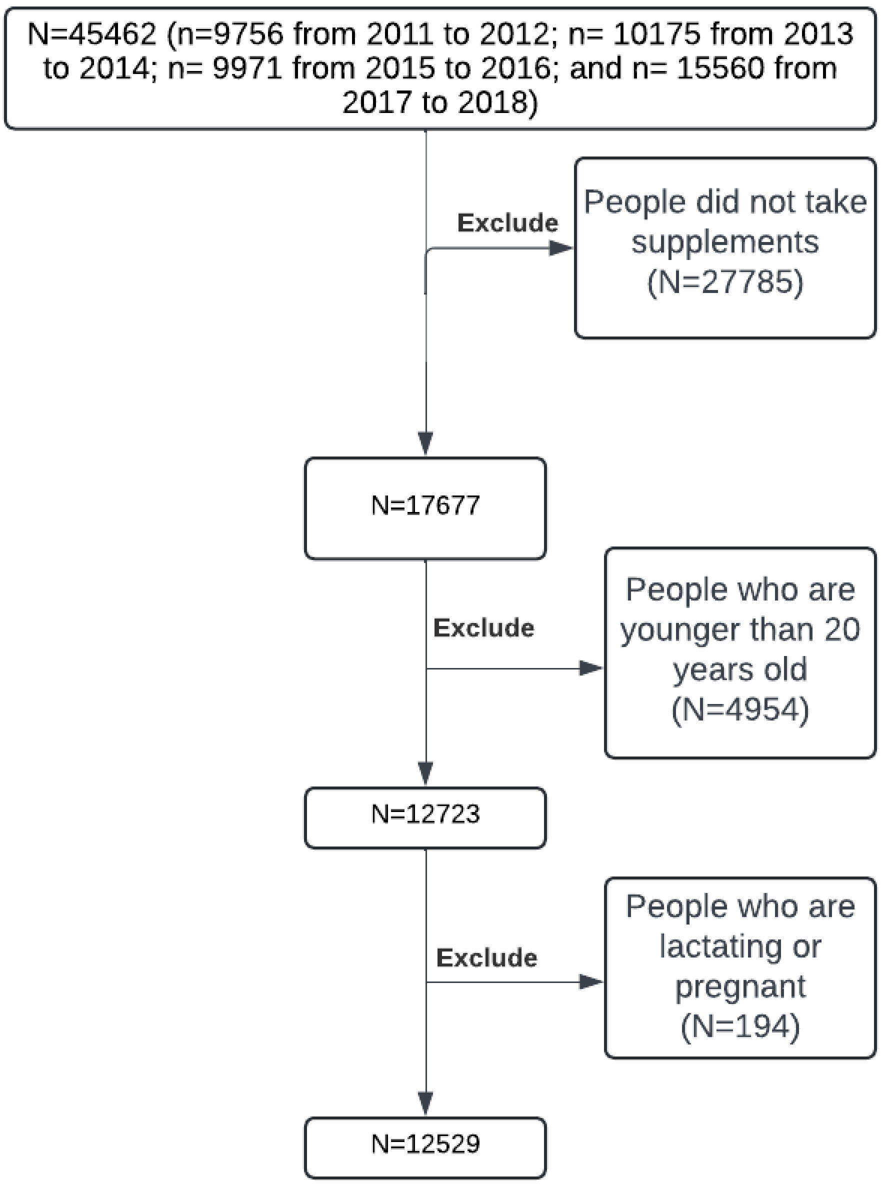

Supplemental Table S2. Prevalence of other reported reasons for dietary supplements use among adults (≥20 Years) by sex and the association between sex and reasons for taking supplements in the United States, 2011-2018

	Percentage (Standard error)		OR (Odds ratio)	
Reason	Male	Female	Unadjusted OR	Adjusted OR
To improve my overall health	38.5 (0.97)	36.28 (0.73)	0.91 (0.83 - 0.99)	**0.9 (0.82 - 1)**
To maintain health (to stay healthy)	36.23 (1.17)	33.55 (1.07)	0.89 (0.78 - 1.01)	0.89 (0.78 - 1.02)
To supplement my diet	19.37 (0.74)	21.05 (0.76)	1.11 (0.99 - 1.24)	**1.15 (1.01 - 1.3)**
To prevent health problems	17.32 (0.85)	16.12 (0.74)	0.92 (0.81 - 1.04)	0.93 (0.82 - 1.06)
To boost immune system	17.2 (0.82)	15.64 (0.69)	0.89 (0.78 - 1.02)	0.9 (0.78 - 1.04)
To get more energy	16.63 (0.68)	19.24 (0.62)	1.19 (1.06 - 1.34)	**1.15 (1.02 - 1.3)**
For heart health, cholesterol	14.42 (0.74)	14.09 (0.61)	0.97 (0.85 - 1.12)	0.92 (0.79 - 1.09)
For bone health	12.39 (0.77)	28.42 (0.81)	2.81 (2.4 - 3.29)	**2.91 (2.41 - 3.51)**
For healthy joints, arthritis	9.69 (0.75)	11.12 (0.57)	1.17 (0.99 - 1.37)	**1.2 (1.01 - 1.44)**
For healthy skin, hair and nails	4.15 (0.35)	13.52 (0.76)	3.61 (2.99 - 4.37)	**3.58 (2.94 - 4.36)**
For muscle health	7.86 (0.53)	5.64 (0.39)	0.7 (0.57 - 0.86)	**0.71 (0.58 - 0.87)**
To maintain blood sugar, diabetes	4.51 (0.32)	4.28 (0.37)	0.95 (0.77 - 1.17)	0.95 (0.75 - 1.2)
For eye health	4.4 (0.32)	5.95 (0.31)	1.38 (1.12 - 1.68)	**1.31 (1.05 - 1.64)**
For relaxation, stress, sleep	4.13 (0.45)	5.02 (0.42)	1.23 (0.93 - 1.62)	**1.29 (1 - 1.66)**
For mental health	3.47 (0.41)	4.62 (0.4)	1.35 (1.04 - 1.76)	**1.36 (1.03 - 1.78)**
For good bowel/colon health	3.24 (0.3)	6.07 (0.53)	1.93 (1.51 - 2.46)	**1.99 (1.54 - 2.57)**
For kidney and bladder health	2.62 (0.31)	2.74 (0.24)	1.05 (0.8 - 1.37)	0.98 (0.73 - 1.33)
For weight loss	2.45 (0.34)	3.39 (0.28)	1.4 (1 - 1.95)	**1.49 (1.08 - 2.06)**
For anemia, such as low iron	2.16 (0.28)	7.05 (0.38)	3.44 (2.61 - 4.53)	**3.31 (2.49 - 4.4)**
For liver health, detoxification	2.01 (0.2)	2 (0.24)	0.99 (0.73 - 1.36)	1.04 (0.74 - 1.46)
Other	1.97 (0.26)	3.48 (0.36)	1.79 (1.34 - 2.41)	**1.87 (1.36 - 2.56)**
Low levels in blood	1.83 (0.27)	3.68 (0.37)	2.05 (1.43 - 2.93)	**2.02 (1.35 - 3.01)**
For weight gain	0.66 (0.11)	0.19 (0.05)	0.29 (0.15 - 0.55)	0.27 (0.13 - 0.53)
For teeth, prevent cavities	0.6 (0.14)	0.96 (0.15)	1.59 (0.88 - 2.88)	1.53 (0.8 - 2.91)
For nervous system health	0.52 (0.13)	0.82 (0.14)	1.57 (0.9 - 2.75)	1.43 (0.81 - 2.51)
Word of mouth, advertisement	0.47 (0.14)	0.52 (0.11)	1.09 (0.58 - 2.06)	0.93 (0.48 - 1.8)
For allergies	0.24 (0.14)	0.29 (0.09)	1.21 (0.38 - 3.86)	1.24 (0.38 - 4.01)
Headaches and dizziness	0.2 (0.09)	0.27 (0.12)	1.36 (0.44 - 4.17)	1.15 (0.43 - 3.04)
For antioxidants	0.15 (0.08)	0.15 (0.08)	1.04 (0.25 - 4.4)	1.06 (0.23 - 4.89)
For thyroid health, gout	0.11 (0.08)	0.71 (0.16)	6.81 (1.37 - 33.86)	**6.57 (1.32 - 32.85)**
For support after surgery	0.1 (0.08)	0.34 (0.11)	3.4 (0.79 - 14.67)	**5.7 (1.64 - 19.85)**
For respiratory health, asthma	0.06 (0.03)	0.08 (0.03)	1.26 (0.38 - 4.18)	1.17 (0.36 - 3.79)

Notes: bold font indicates the statistically significant results in the multivariate-adjusted analysis.

Supplemental Table S3. Prevalence of other reported reasons for dietary supplements use among adults (≥20 Years) by age in the United States, 2011-2018

	Percentage (Standard error)			
Reason	20-39	40-64	65-79	≥80
For good bowel/colon health	3.26 (0.36)	4.99 (0.52)	6.91 (0.88)	4.13 (0.61)
For prostate health	0.48 (0.14)	1.55 (0.25)	2.79 (0.42)	1.89 (0.38)
For mental health	4.28 (0.57)	3.84 (0.45)	4.45 (0.57)	4.41 (0.7)
For teeth, prevent cavities	0.63 (0.14)	0.83 (0.16)	1 (0.24)	0.74 (0.28)
For eye health	1.99 (0.32)	4.4 (0.35)	8.38 (0.71)	16.6 (1.22)
For weight loss	5.22 (0.47)	2.71 (0.31)	1.27 (0.31)	0.42 (0.21)
For pregnancy/breastfeeding	4.6 (0.5)	0.34 (0.12)	0.07 (0.04)	0.14 (0.14)
For anemia, such as low iron	4.55 (0.48)	4.77 (0.38)	4.78 (0.52)	7.89 (0.86)
To maintain blood sugar	3.19 (0.42)	4.59 (0.48)	5.32 (0.58)	5.22 (0.89)
For kidney and bladder health	1.92 (0.28)	2.34 (0.34)	3.97 (0.6)	4.69 (0.66)
For respiratory health, asthma	0.03 (0.03)	0.06 (0.03)	0.16 (0.09)	0.05 (0.05)
For allergies	0.07 (0.05)	0.29 (0.14)	0.57 (0.18)	0.08 (0.08)
To improve digestion	7.73 (0.84)	6.43 (0.55)	8.04 (0.86)	5.09 (0.86)
For menopause, hot flashes	0.36 (0.15)	3.01 (0.39)	0.65 (0.21)	0.09 (0.09)
For muscle health	8.33 (0.7)	5.9 (0.48)	6.73 (0.78)	4.04 (0.68)
For relaxation, stress, sleep	5.47 (0.53)	4.89 (0.49)	3.27 (0.54)	3.14 (0.64)
For nervous system health	0.27 (0.1)	0.66 (0.16)	1.25 (0.29)	1.02 (0.36)
For liver health, detoxification	1.84 (0.28)	2.1 (0.25)	2.31 (0.53)	1.01 (0.43)
For antioxidants	0.08 (0.05)	0.2 (0.1)	0.17 (0.13)	0 (0)
Word of mouth, advertisement	0.3 (0.1)	0.4 (0.1)	0.68 (0.32)	1.54 (0.44)
For thyroid health, gout	0.21 (0.11)	0.51 (0.15)	0.66 (0.34)	0.3 (0.21)
For weight gain	0.8 (0.13)	0.21 (0.08)	0.3 (0.1)	0.29 (0.14)
Low levels in blood	1.38 (0.25)	3.38 (0.38)	3.89 (0.56)	2.43 (0.56)
For support after surgery	0.18 (0.1)	0.34 (0.14)	0.12 (0.07)	0.12 (0.12)
Headaches and dizziness	0.24 (0.14)	0.27 (0.11)	0.19 (0.08)	0.1 (0.1)
Other	1.88 (0.29)	3.12 (0.47)	3.24 (0.48)	3.42 (0.76)

Supplemental Table S4. Prevalence of other reported reasons for dietary supplements use among adults (≥20 Years) by race/ethnicity in the United States, 2011-2018

	Percentage (Standard error)				
	White	Hispanic	Black	Asian	Other
For good bowel/colon health	5.38 (0.45)	3.06 (0.36)	3.98 (0.45)	2.26 (0.32)	5.23 (1.67)
For prostate health	1.47 (0.19)	1.73 (0.32)	1.67 (0.27)	1.06 (0.23)	2.06 (1.22)
For mental health	4.24 (0.4)	3.79 (0.39)	2.86 (0.38)	2.98 (0.49)	7.51 (1.97)
For teeth, prevent cavities	0.76 (0.13)	0.76 (0.17)	1.17 (0.19)	0.71 (0.26)	0.86 (0.34)
For eye health	5.73 (0.27)	3.6 (0.46)	3.8 (0.38)	4.86 (0.64)	5.62 (0.86)
For weight loss	2.58 (0.26)	4.11 (0.64)	4.53 (0.5)	1.85 (0.45)	5.14 (1.23)
For pregnancy/breastfeeding	1.27 (0.18)	2.01 (0.35)	1.65 (0.35)	2.72 (0.36)	0.77 (0.36)
For anemia, such as low iron	4.32 (0.33)	5.55 (0.61)	8.84 (0.76)	4.21 (0.62)	5.71 (1.65)
To maintain blood sugar, diabetes	4.38 (0.37)	4.45 (0.52)	5.21 (0.48)	3.51 (0.45)	3.39 (0.79)
For kidney and bladder health	2.69 (0.25)	2.11 (0.34)	3.52 (0.4)	1.85 (0.52)	3.6 (1.19)
For respiratory health, asthma	0.06 (0.03)	0.19 (0.09)	0.02 (0.02)	0 (0)	0.1 (0.1)
For allergies	0.35 (0.12)	0.08 (0.06)	0.08 (0.06)	0.14 (0.08)	0 (0)
To improve digestion	7.38 (0.56)	6.25 (0.53)	6.15 (0.56)	3.93 (0.46)	8.95 (1.76)
For menopause, hot flashes	1.69 (0.25)	1.57 (0.32)	1.4 (0.25)	0.56 (0.19)	3.16 (2.04)
For muscle health	6.2 (0.38)	8.48 (0.73)	6.59 (0.56)	4.38 (0.62)	12.6 (2.44)
For relaxation, stress, sleep	5.17 (0.41)	4.24 (0.51)	2.38 (0.33)	2.45 (0.37)	3.88 (0.99)
For nervous system health	0.84 (0.14)	0.16 (0.07)	0.46 (0.16)	0.57 (0.19)	0 (0)
For liver health, detoxification	1.88 (0.2)	2.29 (0.44)	2.79 (0.35)	1.66 (0.33)	1.99 (0.71)
For antioxidants	0.17 (0.08)	0.04 (0.04)	0.07 (0.04)	0.25 (0.16)	0.11 (0.11)
Word of mouth, advertisement	0.51 (0.12)	0.23 (0.1)	0.64 (0.2)	0.78 (0.26)	0.39 (0.23)
For thyroid health, gout	0.51 (0.13)	0.29 (0.12)	0.24 (0.11)	0.29 (0.13)	0.38 (0.27)
For weight gain	0.28 (0.07)	0.8 (0.29)	0.9 (0.18)	0.1 (0.07)	0.6 (0.28)
Low levels in blood	2.93 (0.32)	2.42 (0.44)	2.84 (0.35)	2.88 (0.61)	3.08 (0.78)
For support after surgery	0.29 (0.11)	0.05 (0.03)	0.19 (0.06)	0.14 (0.1)	0.1 (0.11)
Headaches and dizziness	0.29 (0.11)	0.08 (0.05)	0 (0)	0.34 (0.14)	0.13 (0.13)
Other	3.11 (0.35)	2.16 (0.4)	2.22 (0.29)	2.12 (0.31)	1.68 (0.65)

Supplemental Table S5. Prevalence of other reported reasons for dietary supplements use among adults (≥20 Years) by education level in the United States, 2011-2018

	Percentage (Standard error)			
Reason	< High school	High School	Some College	≥College
For good bowel/colon health	3.16 (0.55)	3.96 (0.56)	5.07 (0.45)	5.58 (0.58)
For prostate health	1.14 (0.27)	1.64 (0.4)	1.4 (0.24)	1.66 (0.23)
For mental health	3.23 (0.55)	3.64 (0.56)	5.1 (0.51)	3.75 (0.43)
For teeth, prevent cavities	0.88 (0.27)	0.65 (0.18)	0.88 (0.18)	0.79 (0.17)
For eye health	4.82 (0.54)	4.55 (0.44)	5.47 (0.42)	5.62 (0.45)
For weight loss	1.58 (0.32)	2.99 (0.5)	3.8 (0.37)	2.63 (0.41)
For pregnancy/breastfeeding	1.21 (0.26)	1.15 (0.27)	1.25 (0.24)	1.86 (0.25)
For anemia, such as low iron	5.34 (0.58)	4.91 (0.55)	5.01 (0.43)	4.68 (0.47)
To maintain blood sugar, diabetes	5.1 (0.69)	5.75 (0.75)	3.78 (0.4)	3.96 (0.38)
For kidney and bladder health	2.84 (0.51)	2.8 (0.39)	2.98 (0.31)	2.32 (0.41)
For respiratory health, asthma	0.14 (0.07)	0.09 (0.06)	0.1 (0.05)	0.02 (0.02)
For allergies	0.02 (0.02)	0.1 (0.08)	0.38 (0.15)	0.35 (0.19)
To improve digestion	5.03 (0.8)	6.31 (0.78)	7.32 (0.6)	7.72 (0.66)
For menopause, hot flashes	0.86 (0.23)	2.02 (0.54)	1.73 (0.29)	1.58 (0.31)
For muscle health	6.75 (0.79)	6.83 (0.77)	7.47 (0.57)	5.68 (0.56)
For relaxation, stress, sleep	2.57 (0.49)	4.45 (0.7)	4.71 (0.41)	5.25 (0.56)
For nervous system health	0.45 (0.15)	0.7 (0.2)	0.86 (0.18)	0.6 (0.15)
For liver health, detoxification	1.96 (0.3)	1.53 (0.34)	2.3 (0.33)	2.02 (0.25)
For antioxidants	0.04 (0.04)	0.03 (0.03)	0.21 (0.11)	0.2 (0.11)
Word of mouth, advertisement	0.48 (0.15)	0.21 (0.11)	0.79 (0.19)	0.4 (0.13)
For thyroid health, gout	0.18 (0.07)	0.28 (0.18)	0.45 (0.11)	0.61 (0.23)
For weight gain	0.67 (0.22)	0.64 (0.21)	0.29 (0.08)	0.27 (0.12)
Low levels in blood	1.94 (0.44)	3.12 (0.49)	2.7 (0.36)	3.14 (0.36)
For support after surgery	0.15 (0.07)	0.28 (0.19)	0.09 (0.07)	0.38 (0.15)
Headaches and dizziness	0.28 (0.15)	0.14 (0.06)	0.15 (0.09)	0.37 (0.15)
Other	2.52 (0.47)	1.74 (0.28)	2.76 (0.36)	3.55 (0.54)

Supplemental Table S6. Prevalence of other reported reasons for dietary supplements use among adults (≥20 Years) by income level in the United States, 2011-2018

Reason	Percentage (Standard error)		
	< 130%	130% -350%	>350%
For good bowel/colon health	3.44 (0.41)	4.69 (0.45)	5.8 (0.57)
For prostate health	1.2 (0.28)	1.83 (0.29)	1.43 (0.23)
For mental health	3.99 (0.52)	4.53 (0.52)	4.19 (0.49)
For teeth, prevent cavities	1.13 (0.23)	0.78 (0.16)	0.75 (0.18)
For eye health	5.02 (0.51)	5.7 (0.45)	5.2 (0.38)
For weight loss	2.84 (0.38)	3.28 (0.36)	3.2 (0.4)
For pregnancy/breastfeeding	2.14 (0.34)	1.03 (0.18)	1.59 (0.26)
For anemia, such as low iron	7.11 (0.62)	5.25 (0.46)	3.84 (0.41)
To maintain blood sugar, diabetes	4.27 (0.49)	4.44 (0.47)	4.48 (0.47)
For kidney and bladder health	2.77 (0.34)	3.38 (0.41)	2.2 (0.34)
For respiratory health, asthma	0.13 (0.07)	0.16 (0.06)	0 (0)
For allergies	0.04 (0.03)	0.3 (0.15)	0.39 (0.18)
To improve digestion	4.87 (0.52)	7.13 (0.69)	8.12 (0.68)
For menopause, hot flashes	1.37 (0.37)	1.55 (0.3)	1.86 (0.32)
For muscle health	7.58 (0.69)	7.51 (0.45)	5.46 (0.55)
For relaxation, stress, sleep	4.32 (0.55)	4.63 (0.46)	4.57 (0.56)
For nervous system health	0.57 (0.17)	0.88 (0.18)	0.63 (0.17)
For liver health, detoxification	2.32 (0.31)	2.07 (0.23)	1.9 (0.32)
For antioxidants	0.14 (0.09)	0.04 (0.02)	0.26 (0.12)
Word of mouth, advertisement	0.48 (0.18)	0.54 (0.13)	0.39 (0.17)
For thyroid health, gout	0.15 (0.06)	0.45 (0.13)	0.55 (0.18)
For weight gain	0.63 (0.21)	0.55 (0.12)	0.21 (0.09)
Low levels in blood	2.83 (0.4)	2.24 (0.31)	3.31 (0.41)
For support after surgery	0.06 (0.04)	0.17 (0.09)	0.36 (0.15)
Headaches and dizziness	0.35 (0.13)	0.17 (0.1)	0.16 (0.08)
Other	1.92 (0.27)	2.53 (0.29)	3.61 (0.47)

Supplemental Table S7. Logistic regression of age and reasons for taking supplements among adults (≥20 Years) in the United States, 2011-2018

	40-64		65-79		≥80	
	Unadjusted OR	Adjusted OR	Unadjusted OR	Adjusted OR	Unadjusted OR	Adjusted OR
To prevent health problems	1.08 (0.91 - 1.28)	1.07 (0.9 - 1.27)	1.31 (1.08 - 1.58)	**1.35 (1.1 - 1.64)**	0.87 (0.66 - 1.16)	0.92 (0.69 - 1.24)
To improve my overall health	1.09 (0.95 - 1.24)	1.07 (0.92 - 1.23)	1.23 (1.06 - 1.44)	**1.24 (1.05 - 1.45)**	1.04 (0.89 - 1.23)	1.1 (0.92 - 1.32)
To supplement my diet	1 (0.87 - 1.13)	0.96 (0.85 - 1.09)	1.02 (0.85 - 1.21)	0.99 (0.8 - 1.21)	0.93 (0.73 - 1.19)	1.02 (0.78 - 1.32)
To maintain health	1 (0.87 - 1.16)	1 (0.87 - 1.16)	1.12 (0.92 - 1.36)	1.13 (0.92 - 1.38)	1.05 (0.89 - 1.22)	1.08 (0.93 - 1.27)
To boost immune system	0.91 (0.78 - 1.06)	0.92 (0.79 - 1.08)	0.88 (0.73 - 1.06)	0.92 (0.76 - 1.11)	0.53 (0.41 - 0.69)	0.57 (0.44 - 0.75)
For heart health, cholesterol	2.88 (2.32 - 3.58)	**2.84 (2.29 - 3.52)**	5.16 (4.19 - 6.36)	**5.34 (4.33 - 6.58)**	3.22 (2.42 - 4.29)	**3.47 (2.61 - 4.61)**
For healthy joints, arthritis	1.59 (1.33 - 1.91)	**1.52 (1.27 - 1.83)**	2.36 (1.87 - 2.98)	**2.34 (1.8 - 3.06)**	1.94 (1.51 - 2.49)	**2.11 (1.61 - 2.76)**
For healthy skin hair and nails	0.8 (0.67 - 0.96)	**0.78 (0.65 - 0.94)**	0.78 (0.6 - 1.01)	0.83 (0.62 - 1.11)	0.43 (0.31 - 0.59)	**0.47 (0.32 - 0.69)**
For bone health	2.61 (2.17 - 3.13)	**2.7 (2.21 - 3.29)**	5.58 (4.44 - 7)	**6.17 (4.8 - 7.94)**	5.38 (4.45 - 6.5)	**6 (4.84 - 7.44)**
To get more energy	0.84 (0.74 - 0.96)	0.87 (0.75 - 1.02)	0.74 (0.62 - 0.89)	**0.77 (0.63 - 0.94)**	0.58 (0.44 - 0.75)	**0.57 (0.42 - 0.77)**
For good bowel	1.56 (1.14 - 2.13)	**1.49 (1.09 - 2.02)**	2.2 (1.57 - 3.09)	**2.2 (1.58 - 3.06)**	1.28 (0.91 - 1.81)	1.22 (0.84 - 1.77)
For prostate health	3.28 (1.88 - 5.72)	**3.59 (1.9 - 6.76)**	5.97 (3.21 - 11.1)	**7.59 (4.09 - 14.08)**	4.02 (2.01 - 8.03)	**6.57 (3.17 - 13.59)**
For mental health	0.89 (0.65 - 1.22)	0.9 (0.66 - 1.24)	1.04 (0.73 - 1.5)	1.12 (0.76 - 1.65)	1.03 (0.7 - 1.53)	1.02 (0.66 - 1.57)
For teeth	1.33 (0.73 - 2.41)	1.39 (0.75 - 2.6)	1.6 (0.82 - 3.12)	1.83 (0.99 - 3.4)	1.18 (0.48 - 2.91)	1.2 (0.44 - 3.24)
For eye health	2.27 (1.55 - 3.32)	**2.35 (1.62 - 3.41)**	4.51 (3.17 - 6.4)	**4.88 (3.45 - 6.91)**	9.81 (6.67 - 14.4)	10.06 (6.8 - 14.87)
For weight loss	0.51 (0.38 - 0.68)	**0.47 (0.35 - 0.64)**	0.23 (0.14 - 0.39)	**0.24 (0.14 - 0.4)**	0.08 (0.03 - 0.21)	**0.05 (0.02 - 0.17)**
For pregnancy/breastfeeding	0.07 (0.04 - 0.14)	**0.06 (0.03 - 0.12)**	0.01 (0 - 0.05)	**0.01 (0 - 0.06)**	0.03 (0 - 0.24)	**0.03 (0 - 0.24)**
For anemia	1.05 (0.81 - 1.37)	1.17 (0.87 - 1.57)	1.05 (0.77 - 1.45)	1.15 (0.81 - 1.62)	1.8 (1.31 - 2.48)	**1.87 (1.28 - 2.74)**
To maintain blood sugar	1.46 (1.02 - 2.09)	1.44 (0.99 - 2.09)	1.7 (1.21 - 2.41)	**1.67 (1.13 - 2.48)**	1.67 (1.12 - 2.48)	**1.51 (1 - 2.28)**
For kidney and bladder	1.23 (0.81 - 1.88)	1.24 (0.8 - 1.93)	2.12 (1.33 - 3.38)	**2.11 (1.29 - 3.46)**	2.52 (1.64 - 3.89)	**2.62 (1.67 - 4.11)**
For respiratory health	2.23 (0.26 - 18.91)	2.94 (0.37 - 23.23)	5.96 (0.65 - 54.68)	7.38 (0.94 - 58.17)	1.96 (0.12 - 31.65)	2.03 (0.17 - 24.66)
For allergies	4.09 (0.79 - 21.24)	3.75 (0.71 - 19.67)	7.99 (1.86 - 34.39)	**7.3 (1.69 - 31.53)**	1.05 (0.1 - 11.15)	1.14 (0.1 - 12.38)
To improve digestion	0.82 (0.61 - 1.1)	0.78 (0.58 - 1.04)	1.04 (0.76 - 1.43)	1.03 (0.75 - 1.42)	0.64 (0.42 - 0.98)	0.68 (0.42 - 1.1)
For menopause	8.57 (3.62 - 20.32)	**8.11 (3.27 - 20.13)**	1.82 (0.67 - 4.97)	1.68 (0.56 - 4.99)	0.26 (0.03 - 2.16)	0.25 (0.03 - 2.18)
For muscle health	0.69 (0.54 - 0.87)	**0.74 (0.59 - 0.93)**	0.79 (0.58 - 1.08)	0.82 (0.59 - 1.14)	0.46 (0.32 - 0.67)	**0.52 (0.37 - 0.75)**
For relax, stress, sleep	0.89 (0.66 - 1.19)	0.84 (0.62 - 1.13)	0.58 (0.41 - 0.84)	**0.59 (0.41 - 0.85)**	0.56 (0.36 - 0.88)	**0.58 (0.36 - 0.95)**
For nervous system	2.46 (1.05 - 5.78)	2.33 (0.98 - 5.54)	4.65 (1.89 - 11.45)	**4.4 (1.77 - 10.96)**	3.8 (1.45 - 9.98)	**3.35 (1.28 - 8.75)**
For liver health	1.14 (0.77 - 1.71)	1.25 (0.83 - 1.88)	1.26 (0.72 - 2.2)	1.42 (0.83 - 2.43)	0.55 (0.22 - 1.34)	0.56 (0.25 - 1.25)
For antioxidants	2.56 (0.56 - 11.67)	2.39 (0.6 - 9.51)	2.13 (0.32 - 14.15)	2.25 (0.33 - 15.1)	0 (0 - 0)	0 (0 - 0)
Word of mouth	1.32 (0.58 - 3.02)	1.09 (0.44 - 2.7)	2.24 (0.78 - 6.42)	2.19 (0.7 - 6.81)	5.14 (2.08 - 12.73)	**5.2 (2.04 - 13.29)**
For thyroid health, gout	2.4 (0.76 - 7.57)	2.11 (0.65 - 6.77)	3.11 (0.73 - 13.16)	2.55 (0.54 - 12.03)	1.4 (0.24 - 8.2)	1.49 (0.26 - 8.42)
For weight gain	0.26 (0.11 - 0.63)	0.3 (0.12 - 0.75)	0.37 (0.18 - 0.76)	0.47 (0.22 - 1.02)	0.36 (0.13 - 0.96)	0.48 (0.16 - 1.42)
Low levels in blood	2.49 (1.69 - 3.67)	**2.5 (1.67 - 3.76)**	2.89 (1.85 - 4.5)	**3.06 (1.86 - 5.04)**	1.78 (0.96 - 3.29)	**1.98 (1 - 3.91)**
For support after surgery	1.92 (0.67 - 5.54)	1.44 (0.42 - 4.96)	0.69 (0.14 - 3.41)	0.58 (0.11 - 2.99)	0.71 (0.08 - 6.69)	0.64 (0.06 - 6.48)
Headaches and dizziness	1.15 (0.48 - 2.76)	**5.06 (1.39 - 18.39)**	0.78 (0.18 - 3.27)	3.37 (0.58 - 19.45)	0.43 (0.04 - 4.34)	1.62 (0.13 - 19.8)
Other	1.68 (1.06 - 2.65)	**1.61 (1 - 2.6)**	1.75 (1.11 - 2.75)	**1.67 (1.02 - 2.73)**	1.85 (1.09 - 3.13)	**1.86 (1.06 - 3.25)**

Notes: bold font indicates the statistically significant results in the multivariate-adjusted analysis; OR: odds ratio.

Supplemental Table S8. The association between race/ethnicity and reasons for taking supplements among adults (≥20 Years) in the United States, 2011-2018

	Hispanic		Black		Asian		Other races	
Reason	Unadjusted OR	Adjusted OR	Unadjusted OR	Adjusted OR	Unadjusted OR	Adjusted OR	Unadjusted OR	Adjusted OR
To prevent health problems	0.74 (0.6 - 0.9)	1.08 (0.86 - 1.35)	0.83 (0.68 - 1.02)	1.03 (0.8 - 1.29)	0.73 (0.6 - 0.87)	**0.78 (0.6 - 1)**	1 (0.7 - 1.44)	1.14 (0.77 - 1.7)
To improve overall health	0.65 (0.57 - 0.74)	**1.26 (1.09 - 1.44)**	0.82 (0.74 - 0.91)	1.14 (0.99 - 1.3)	0.85 (0.7 - 0.98)	1.14 (0.96 - 1.35)	0.77 (0.6 - 0.98)	0.97 (0.74 - 1.26)
To supplement diet	0.73 (0.63 - 0.85)	1.14 (0.97 - 1.35)	0.85 (0.71 - 1.01)	1.0 (0.85 - 1.28)	0.99 (0.8 - 1.17)	1.14 (0.92 - 1.42)	1.04 (0.77 - 1.4)	1.24 (0.89 - 1.73)
To maintain health	0.7 (0.61 - 0.8)	**1.22 (1.05 - 1.41)**	0.81 (0.7 - 0.93)	1.1 (0.95 - 1.29)	1.06 (0.95 - 1.2)	**1.22 (1.04 - 1.43)**	0.96 (0.71 - 1.31)	1.26 (0.9 - 1.74)
To boost immune system	0.91 (0.75 - 1.11)	0.96 (0.79 - 1.18)	1.09 (0.94 - 1.27)	1.11 (0.9 - 1.35)	0.73 (0.6 - 0.9)	0.7 (0.56 - 0.89)	1.65 (1.13 - 2.42)	1.54 (0.97 - 2.46)
For heart health, cholesterol	0.66 (0.53 - 0.83)	1.04 (0.82 - 1.33)	0.78 (0.66 - 0.92)	0.95 (0.76 - 1.2)	0.92 (0.77 - 1.1)	1.19 (0.93 - 1.53)	1.12 (0.81 - 1.55)	1.36 (0.9 - 2.05)
For healthy joints, arthritis	0.79 (0.65 - 0.96)	0.99 (0.81 - 1.2)	0.84 (0.67 - 1.04)	0.95 (0.75 - 1.2)	0.96 (0.77 - 1.2)	1.09 (0.86 - 1.38)	1.14 (0.82 - 1.59)	1.3 (0.84 - 2.01)
For healthy skin, hair, nails	1.35 (1.12 - 1.63)	**0.7 (0.56 - 0.87)**	1.53 (1.21 - 1.92)	1.03 (0.8 - 1.33)	1.18 (0.9 - 1.51)	0.83 (0.65 - 1.07)	1.07 (0.71 - 1.61)	0.8 (0.49 - 1.32)
For bone health	0.92 (0.8 - 1.06)	**0.77 (0.65 - 0.92)**	0.86 (0.74 - 0.99)	**0.76 (0.6 - 0.96)**	1.08 (0.9 - 1.28)	0.93 (0.75 - 1.16)	1.07 (0.75 - 1.54)	1.19 (0.77 - 1.84)
To get more energy	1.68 (1.47 - 1.92)	0.67 (0.58 - 0.77)	1.91 (1.65 - 2.21)	**1.18 (1.0 - 1.39)**	0.75 (0.63 - 0.9)	**0.51 (0.43 - 0.61)**	1.35 (0.97 - 1.89)	0.79 (0.57 - 1.07)
good bowel	0.55 (0.41 - 0.74)	1.35 (0.98 - 1.85)	0.73 (0.55 - 0.97)	1.0 (0.69 - 1.49)	0.4 (0.29 - 0.56)	**0.59 (0.42 - 0.83)**	0.97 (0.5 - 1.9)	1.46 (0.68 - 3.15)
prostate health	1.18 (0.81 - 1.73)	**0.52 (0.33 - 0.82)**	1.14 (0.73 - 1.78)	0.9 (0.48 - 1.53)	0.7 (0.45 - 1.14)	0.53 (0.28 - 1.02)	1.41 (0.46 - 4.34)	0.75 (0.23 - 2.39)
mental health	0.89 (0.66 - 1.2)	1.01 (0.73 - 1.4)	0.66 (0.47 - 0.94)	0.7 (0.51 - 1)	0.69 (0.5 - 0.98)	0.72 (0.47 - 1.13)	1.83 (1.05 - 3.2)	**1.93 (1.04 - 3.56)**
teeth	0.99 (0.54 - 1.83)	**1.04 (0.55 - 1.97)**	1.54 (0.96 - 2.47)	1.3 (0.69 - 2.55)	0.9 (0.42 - 2.08)	0.94 (0.36 - 2.49)	1.12 (0.5 - 2.53)	1.01 (0.32 - 3.14)
eye health	0.61 (0.46 - 0.81)	1.07 (0.78 - 1.46)	0.65 (0.51 - 0.83)	0.8 (0.54 - 1.18)	0.8 (0.62 - 1.13)	1.16 (0.8 - 1.68)	0.98 (0.71 - 1.35)	1.25 (0.74 - 2.12)
weight loss	1.62 (1.09 - 2.4)	**0.66 (0.45 - 0.97)**	1.79 (1.32 - 2.43)	1.1 (0.71 - 1.65)	0.7 (0.43 - 1.18)	**0.45 (0.25 - 0.8)**	2.05 (1.18 - 3.54)	1.21 (0.72 - 2.05)
pregnancy/breastfeeding	1.59 (1.05 - 2.41)	0.87 (0.57 - 1.35)	1.3 (0.8 - 2.11)	0.9 (0.54 - 1.5)	2.2 (1.49 - 3.16)	1.32 (0.76 - 2.32)	0.6 (0.21 - 1.67)	0.42 (0.14 - 1.3)
anemia, such as low iron	1.3 (1 - 1.7)	0.77 (0.58 - 1.03)	2.15 (1.65 - 2.8)	**1.5 (1.12 - 2)**	1 (0.69 - 1.39)	0.69 (0.46 - 1.04)	1.34 (0.72 - 2.5)	1.14 (0.62 - 2.1)
diabetes	1.02 (0.75 - 1.37)	0.93 (0.67 - 1.28)	1.2 (0.94 - 1.53)	1.2 (0.84 - 1.65)	0.8 (0.57 - 1.1)	0.76 (0.53 - 1.09)	0.77 (0.46 - 1.27)	0.7 (0.38 - 1.29)
kidney and bladder	0.78 (0.54 - 1.12)	1.04 (0.69 - 1.55)	1.32 (0.97 - 1.79)	1.4 (0.94 - 2.15)	0.7 (0.41 - 1.13)	0.88 (0.48 - 1.62)	1.35 (0.68 - 2.7)	1.51 (0.79 - 2.88)
respiratory health,	2.97 (0.8 - 10.58)	0.39 (0.12 - 1.31)	0.39 (0.05 - 3.21)	**0.1 (0.01 - 0.48)**	0 (0 - 0)	0 (0 - 0)	1.6 (0.19 - 13.36)	0.48 (0.05 - 4.29)
allergies	0.24 (0.05 - 1.15)	2.09 (0.43 - 10.17)	0.23 (0.05 - 1.06)	0.7 (0.09 - 4.63)	0.4 (0.1 - 1.46)	1.05 (0.16 - 6.78)	0 (0 - 0)	0 (0 - 0)
improve digestion	0.84 (0.65 - 1.07)	1.01 (0.75 - 1.38)	0.82 (0.64 - 1.06)	0.9 (0.71 - 1.18)	0.5 (0.38 - 0.69)	**0.5 (0.36 - 0.68)**	1.23 (0.81 - 1.87)	1.17 (0.71 - 1.95)
menopause, hot flashes	0.92 (0.56 - 1.53)	0.96 (0.53 - 1.72)	0.83 (0.52 - 1.3)	0.7 (0.37 - 1.21)	0.3 (0.16 - 0.68)	**0.3 (0.14 - 0.66)**	1.89 (0.48 - 7.45)	2.31 (0.54 - 9.91)
muscle health	1.4 (1.11 - 1.77)	0.77 (0.59 - 1.02)	1.07 (0.88 - 1.29)	0.8 (0.57 - 1.07)	0.7 (0.49 - 0.97)	**0.55 (0.38 - 0.81)**	2.18 (1.43 - 3.33)	1.43 (0.84 - 2.43)
relaxation, stress, sleep	0.81 (0.61 - 1.08)	1.14 (0.85 - 1.53)	0.45 (0.33 - 0.61)	**0.6 (0.38 - 0.8)**	0.5 (0.33 - 0.64)	**0.58 (0.39 - 0.85)**	0.74 (0.44 - 1.25)	0.86 (0.47 - 1.58)
nervous system health	0.19 (0.08 - 0.46)	**4.89 (1.87 - 12.8)**	0.54 (0.27 - 1.1)	3.1 (0.9 - 10.55)	0.7 (0.33 - 1.39)	4.66 (1.6 - 13.53)	0 (0 - 0)	0 (0 - 0)
liver health,	1.22 (0.79 - 1.88)	0.8 (0.5 - 1.28)	1.5 (1.07 - 2.09)	1.2 (0.7 - 1.91)	0.9 (0.57 - 1.34)	0.72 (0.39 - 1.33)	1.06 (0.5 - 2.23)	0.76 (0.31 - 1.88)
antioxidants	0.21 (0.03 - 1.74)	3.05 (0.48 - 19.53)	0.42 (0.1 - 1.81)	1.4 (0.16 - 12.9)	1.5 (0.31 - 6.88)	4.96 (0.49 - 50.5)	0.64 (0.07 - 5.56)	2.26 (0.16 - 31.54)
advertisement	0.44 (0.17 - 1.13)	1.8 (0.59 - 5.48)	1.27 (0.55 - 2.91)	2.6 (0.8 - 8.72)	1.6 (0.84 - 2.88)	**3.96 (1.23 - 12.8)**	0.78 (0.21 - 2.84)	1.13 (0.21 - 6.08)
thyroid health, gout	0.57 (0.21 - 1.56)	1.01 (0.39 - 2.62)	0.47 (0.17 - 1.33)	0.6 (0.2 - 2.05)	0.6 (0.2 - 1.55)	0.63 (0.19 - 2.13)	0.73 (0.19 - 2.81)	1.1 (0.26 - 4.71)
weight gain	2.87 (1 - 8.23)	0.5 (0.17 - 1.48)	3.24 (1.78 - 5.91)	1.5 (0.64 - 3.6)	0.4 (0.08 - 1.66)	**0.18 (0.04 - 0.91)**	2.17 (0.82 - 5.78)	0.56 (0.13 - 2.38)
Low levels in blood	0.82 (0.54 - 1.26)	1.06 (0.67 - 1.67)	0.97 (0.71 - 1.32)	1.0 (0.66 - 1.65)	1 (0.61 - 1.59)	1.22 (0.64 - 2.3)	1.05 (0.61 - 1.82)	1.35 (0.67 - 2.74)
support after surgery	0.16 (0.04 - 0.69)	**4.28 (1.06 - 17.3)**	0.65 (0.24 - 1.73)	3.7 (0.9 - 14.7)	0.5 (0.11 - 2.32)	1.93 (0.29 - 12.8)	0.36 (0.05 - 2.95)	2.34 (0.21 - 26.45)
Headaches and dizziness	0.26 (0.08 - 0.86)	**6.27 (1.08 - 36.3)**	0 (0 - 0)	0 (0 - 0)	1.2 (0.4 - 3.46)	**6.66 (1.11 - 40.0)**	0.44 (0.05 - 3.73)	3.35 (0.27 - 41.67)
Other	0.69 (0.45 - 1.05)	0.94 (0.57 - 1.56)	0.71 (0.5 - 1)	0.7 (0.45 - 1.24)	0.7 (0.45 - 1.02)	0.74 (0.29 - 1.89)	0.53 (0.24 - 1.19)	0.57 (0.33 - 1)

Notes: bold font indicates the statistically significant results in the multivariate-adjusted analysis; OR: odds ratio.

Supplemental Table S9. The association between education level and reasons for taking supplements among adults (≥20 Years) in the United States, 2011-2018

	High school		Some college		≥College	
Reason	Unadjusted OR	Adjusted OR	Unadjusted OR	Adjusted OR	Unadjusted OR	Adjusted OR
To prevent health problems	1.21 (0.92 - 1.59)	1.08 (0.81 - 1.44)	1.56 (1.19 - 2.06)	**1.44 (1.08 - 1.91)**	1.95 (1.53 - 2.48)	**1.78 (1.35 - 2.34)**
To improve overall health	1.68 (1.39 - 2.02)	**1.52 (1.26 - 1.85)**	1.9 (1.65 - 2.19)	**1.74 (1.47 - 2.06)**	1.94 (1.62 - 2.32)	**1.72 (1.37 - 2.17)**
To supplement diet	1.26 (1.03 - 1.53)	1.2 (0.97 - 1.47)	1.49 (1.26 - 1.77)	**1.38 (1.16 - 1.65)**	1.86 (1.54 - 2.24)	**1.6 (1.29 - 1.97)**
To maintain health	1.26 (1.02 - 1.56)	1.14 (0.9 - 1.44)	1.5 (1.21 - 1.86)	**1.4 (1.1 - 1.78)**	2.04 (1.7 - 2.44)	**1.82 (1.47 - 2.24)**
To boost immune system	1.26 (0.92 - 1.71)	1.21 (0.89 - 1.65)	1.64 (1.2 - 2.24)	**1.57 (1.13 - 2.17)**	1.68 (1.28 - 2.22)	**1.67 (1.25 - 2.24)**
For heart health, cholesterol	1.41 (1.08 - 1.84)	1.45 (1.09 - 1.94)	1.29 (0.97 - 1.72)	**1.36 (1 - 1.84)**	1.31 (0.98 - 1.75)	**1.41 (1.05 - 1.9)**
For healthy joints, arthritis	1.21 (0.91 - 1.6)	1.18 (0.88 - 1.58)	1.39 (1.13 - 1.72)	**1.31 (1.02 - 1.69)**	1.26 (1.01 - 1.58)	1.18 (0.92 - 1.51)
For healthy skin, hair, nails	1.43 (1.02 - 2.02)	**1.45 (1.03 - 2.05)**	1.68 (1.31 - 2.15)	**1.6 (1.19 - 2.15)**	1.46 (1.19 - 1.79)	**1.49 (1.09 - 2.03)**
For bone health	1.02 (0.84 - 1.23)	1.13 (0.91 - 1.41)	0.87 (0.74 - 1.02)	1.02 (0.82 - 1.27)	1.01 (0.85 - 1.2)	**1.32 (1.03 - 1.69)**
To get more energy	0.95 (0.78 - 1.15)	0.99 (0.8 - 1.24)	0.93 (0.77 - 1.12)	0.97 (0.77 - 1.22)	0.74 (0.61 - 0.9)	0.88 (0.66 - 1.17)
For good bowel/colon health	1.26 (0.81 - 1.96)	0.99 (0.62 - 1.6)	1.64 (1.12 - 2.4)	1.32 (0.85 - 2.05)	1.81 (1.2 - 2.75)	1.41 (0.85 - 2.35)
For prostate health	1.44 (0.8 - 2.61)	1.8 (0.95 - 3.4)	1.24 (0.74 - 2.07)	**1.95 (1.06 - 3.56)**	1.46 (0.85 - 2.52)	**2.12 (1.06 - 4.27)**
For mental health	1.13 (0.72 - 1.8)	1.04 (0.65 - 1.67)	1.61 (1.11 - 2.34)	**1.47 (1 - 2.18)**	1.17 (0.76 - 1.8)	1.14 (0.7 - 1.86)
For teeth, prevent cavities	0.73 (0.3 - 1.78)	0.82 (0.27 - 2.51)	0.99 (0.46 - 2.14)	1.2 (0.43 - 3.33)	0.9 (0.47 - 1.7)	1.23 (0.44 - 3.44)
For eye health	0.94 (0.7 - 1.27)	1.06 (0.77 - 1.46)	1.14 (0.87 - 1.51)	**1.46 (1.07 - 1.99)**	1.17 (0.86 - 1.6)	**1.71 (1.18 - 2.48)**
For weight loss	1.93 (1.11 - 3.34)	1.66 (0.96 - 2.88)	2.47 (1.53 - 3.98)	**1.88 (1.15 - 3.06)**	1.69 (0.99 - 2.88)	1.22 (0.69 - 2.17)
pregnancy/breastfeeding	0.95 (0.51 - 1.78)	1.24 (0.6 - 2.59)	1.03 (0.61 - 1.75)	0.85 (0.48 - 1.5)	1.55 (0.92 - 2.59)	1.18 (0.64 - 2.18)
For anemia, such as low iron	0.92 (0.68 - 1.23)	1.1 (0.8 - 1.5)	0.93 (0.7 - 1.25)	1.15 (0.86 - 1.55)	0.87 (0.63 - 1.21)	**1.44 (1.01 - 2.07)**
To maintain blood sugar	1.13 (0.77 - 1.68)	1.17 (0.76 - 1.8)	0.73 (0.53 - 1.01)	0.73 (0.47 - 1.13)	0.77 (0.57 - 1.03)	0.74 (0.47 - 1.15)
For kidney and bladder	0.99 (0.66 - 1.48)	1.04 (0.68 - 1.61)	1.05 (0.7 - 1.57)	1.26 (0.8 - 1.99)	0.81 (0.51 - 1.3)	1 (0.57 - 1.74)
For respiratory health	0.64 (0.11 - 3.62)	0.92 (0.16 - 5.25)	0.71 (0.17 - 2.95)	1.31 (0.34 - 5.04)	0.15 (0.02 - 0.93)	0.6 (0.07 - 5.21)
For allergies	4.67 (0.37 - 58.96)	3.15 (0.25 - 39.4)	16.8 (2.0 - 138.4)	**11.3 (1.46 - 87.1)**	15.4 (1.7 - 144.3)	**9.2 (1.16 - 73.63)**
To improve digestion	1.27 (0.88 - 1.84)	1.07 (0.7 - 1.64)	1.49 (1.06 - 2.1)	1.12 (0.77 - 1.64)	1.58 (1.13 - 2.21)	1.16 (0.76 - 1.79)
For menopause, hot flashes	2.38 (1.24 - 4.58)	1.74 (0.93 - 3.25)	2.04 (1.06 - 3.94)	1.34 (0.68 - 2.64)	1.86 (0.96 - 3.6)	1.24 (0.61 - 2.51)
For muscle health	1.01 (0.7 - 1.46)	0.99 (0.69 - 1.43)	1.11 (0.82 - 1.52)	1.06 (0.77 - 1.46)	0.83 (0.6 - 1.15)	0.93 (0.62 - 1.38)
For relaxation, stress, sleep	1.76 (1.08 - 2.87)	1.58 (0.98 - 2.57)	1.87 (1.28 - 2.73)	1.52 (0.98 - 2.35)	2.09 (1.38 - 3.17)	**1.77 (1.13 - 2.77)**
For nervous system health	1.57 (0.65 - 3.78)	1.85 (0.74 - 4.58)	1.94 (0.91 - 4.11)	**2.46 (1.07 - 5.65)**	1.34 (0.58 - 3.09)	1.51 (0.58 - 3.91)
For liver health	0.78 (0.46 - 1.31)	0.67 (0.39 - 1.17)	1.18 (0.78 - 1.77)	1.21 (0.79 - 1.88)	1.03 (0.72 - 1.47)	1.22 (0.78 - 1.92)
For antioxidants	0.7 (0.04 - 11.19)	0.6 (0.04 - 8.8)	5.32 (0.6 - 47.27)	4.03 (0.5 - 31.26)	4.95 (0.5 - 46.5)	2.77 (0.3 - 25.39)
advertisement	0.45 (0.14 - 1.46)	0.62 (0.16 - 2.37)	1.67 (0.81 - 3.45)	2.15 (0.93 - 5)	0.83 (0.32 - 2.14)	1.45 (0.55 - 3.77)
For thyroid health, gout	1.55 (0.35 - 6.97)	1.2 (0.29 - 5.07)	2.52 (1 - 6.35)	1.58 (0.61 - 4.07)	3.42 (1.18 - 9.93)	2.32 (0.76 - 7.1)
For weight gain	0.97 (0.4 - 2.34)	1.22 (0.43 - 3.48)	0.43 (0.18 - 1.04)	0.59 (0.21 - 1.67)	0.4 (0.13 - 1.25)	0.8 (0.27 - 2.32)
Low levels in blood	1.62 (0.98 - 2.7)	1.75 (0.96 - 3.18)	1.4 (0.78 - 2.51)	1.68 (0.88 - 3.21)	1.64 (1 - 2.68)	1.74 (0.92 - 3.29)
For support after surgery	1.85 (0.37 - 9.24)	0.75 (0.19 - 2.86)	0.58 (0.11 - 3.13)	0.26 (0.05 - 1.25)	2.54 (0.76 - 8.49)	0.92 (0.28 - 3.03)
Headaches and dizziness	0.48 (0.15 - 1.59)	0.42 (0.09 - 1.91)	0.52 (0.1 - 2.69)	0.57 (0.11 - 3)	1.31 (0.47 - 3.63)	1.04 (0.39 - 2.8)
Other	0.69 (0.41 - 1.16)	0.57 (0.33 - 1.01)	1.1 (0.71 - 1.69)	0.93 (0.58 - 1.47)	1.42 (0.91 - 2.23)	1.12 (0.68 - 1.87)

Notes: bold font indicates the statistically significant results in the multivariate-adjusted analysis; OR: odds ratio.

Supplemental Table S10. The association between income level and reasons for taking supplements among adults (≥20 Years) in the United States, 2011-2018

	130% -350%		>350%	
Reason	Unadjusted OR	Adjusted OR	Unadjusted OR	Adjusted OR
To prevent health problems	1.44 (1.14 - 1.81)	**1.61 (1.34 - 1.94)**	1.29 (1.01 - 1.64)	**1.25 (1.01 - 1.54)**
To improve my overall health	1.35 (1.15 - 1.58)	**1.46 (1.26 - 1.68)**	1.2 (1.01 - 1.41)	**1.19 (1 - 1.42)**
To supplement my diet	1.1 (0.94 - 1.29)	**1.42 (1.21 - 1.67)**	1.03 (0.87 - 1.21)	**1.22 (1.03 - 1.44)**
To maintain health	1.42 (1.22 - 1.64)	**1.79 (1.53 - 2.08)**	1.26 (1.08 - 1.47)	**1.36 (1.16 - 1.6)**
To boost immune system	1.32 (1.09 - 1.6)	1.23 (0.99 - 1.52)	1.26 (1.03 - 1.53)	1.07 (0.86 - 1.33)
For heart health, cholesterol	1.4 (1.14 - 1.72)	**1.36 (1.09 - 1.69)**	1.18 (0.96 - 1.45)	1.11 (0.89 - 1.39)
For healthy joints, arthritis	1.17 (0.92 - 1.48)	**1.41 (1.12 - 1.78)**	1.07 (0.82 - 1.39)	**1.34 (1.04 - 1.73)**
For healthy skin, hair and nails	0.88 (0.72 - 1.08)	0.87 (0.7 - 1.09)	0.95 (0.79 - 1.16)	1.01 (0.77 - 1.33)
For bone health	1.1 (0.93 - 1.29)	1.15 (0.98 - 1.35)	0.99 (0.83 - 1.19)	1.12 (0.92 - 1.35)
To get more energy	0.99 (0.84 - 1.16)	**0.74 (0.61 - 0.89)**	1.11 (0.95 - 1.31)	0.9 (0.72 - 1.14)
For good bowel/colon health	1.38 (0.96 - 1.97)	**1.73 (1.25 - 2.38)**	1.23 (0.85 - 1.8)	1.43 (0.98 - 2.08)
For prostate health	1.53 (0.97 - 2.42)	1.19 (0.64 - 2.22)	1.15 (0.71 - 1.85)	0.77 (0.42 - 1.44)
For mental health	1.14 (0.81 - 1.61)	1.05 (0.76 - 1.45)	1.11 (0.79 - 1.55)	1.07 (0.76 - 1.49)
For teeth, prevent cavities	0.69 (0.38 - 1.26)	0.67 (0.34 - 1.32)	0.66 (0.35 - 1.24)	0.6 (0.27 - 1.37)
For eye health	1.14 (0.86 - 1.52)	1.04 (0.79 - 1.36)	0.9 (0.68 - 1.21)	0.82 (0.62 - 1.1)
For weight loss	1.16 (0.79 - 1.69)	1.13 (0.77 - 1.66)	1.4 (0.97 - 2.01)	**1.61 (1.08 - 2.38)**
For pregnancy/breastfeeding	0.48 (0.31 - 0.74)	0.74 (0.48 - 1.14)	0.67 (0.42 - 1.05)	1.24 (0.74 - 2.06)
For anemia, such as low iron	0.72 (0.56 - 0.93)	**0.52 (0.38 - 0.71)**	0.75 (0.58 - 0.97)	**0.56 (0.4 - 0.8)**
To maintain blood sugar	1.04 (0.76 - 1.43)	1.05 (0.75 - 1.47)	1.06 (0.76 - 1.5)	1.19 (0.81 - 1.76)
For kidney and bladder	1.23 (0.86 - 1.76)	0.79 (0.51 - 1.22)	1.15 (0.79 - 1.67)	0.83 (0.54 - 1.27)
For respiratory health,	1.24 (0.32 - 4.71)	0 (0 - 0)	1.21 (0.25 - 5.86)	0 (0 - 0)
For allergies	7.59 (1.35 - 42.53)	**9.84 (1.79 - 54.19)**	4.87 (0.9 - 26.44)	4.71 (0.98 - 22.74)
To improve digestion	1.5 (1.13 - 1.98)	**1.73 (1.33 - 2.24)**	1.5 (1.12 - 2.02)	**1.75 (1.32 - 2.32)**
For menopause, hot flashes	1.14 (0.6 - 2.17)	1.37 (0.72 - 2.61)	1.2 (0.66 - 2.18)	1.34 (0.73 - 2.45)
For muscle health	0.99 (0.78 - 1.25)	**0.7 (0.56 - 0.88)**	1.03 (0.8 - 1.31)	0.77 (0.58 - 1.03)
For relaxation, stress, sleep	1.08 (0.76 - 1.52)	1.06 (0.74 - 1.52)	1.02 (0.71 - 1.46)	0.9 (0.61 - 1.32)
For nervous system health	1.55 (0.82 - 2.91)	1.1 (0.5 - 2.41)	1.17 (0.63 - 2.17)	0.85 (0.4 - 1.81)
For liver health	0.89 (0.64 - 1.24)	0.82 (0.53 - 1.26)	0.88 (0.62 - 1.25)	0.73 (0.45 - 1.21)
For antioxidants	0.26 (0.05 - 1.4)	1.81 (0.41 - 7.97)	0.2 (0.04 - 1.1)	1.04 (0.27 - 3.99)
Word of mouth	1.13 (0.46 - 2.77)	0.81 (0.25 - 2.61)	0.94 (0.37 - 2.39)	0.71 (0.22 - 2.31)
For thyroid health, gout	2.94 (1.19 - 7.23)	**3.59 (1.28 - 10.06)**	2.56 (1.08 - 6.05)	2.6 (0.95 - 7.11)
For weight gain	0.87 (0.41 - 1.85)	0.33 (0.08 - 1.33)	1 (0.48 - 2.09)	0.49 (0.16 - 1.5)
Low levels in blood	0.79 (0.52 - 1.21)	1.18 (0.81 - 1.71)	0.7 (0.46 - 1.05)	1 (0.66 - 1.52)
For support after surgery	2.83 (0.64 - 12.63)	**5.88 (1.34 - 25.85)**	2.95 (0.77 - 11.36)	**4.93 (1.08 - 22.56)**
Headaches and dizziness	0.48 (0.12 - 1.86)	0.47 (0.18 - 1.19)	0.39 (0.1 - 1.55)	0.24 (0.1 - 0.61)
Other	1.33 (0.96 - 1.84)	**1.92 (1.35 - 2.73)**	1.28 (0.91 - 1.79)	**1.64 (1.08 - 2.49)**

Notes: bold font indicates the statistically significant results in the multivariate-adjusted analysis; OR: odds ratio.
